# Foxp3 Interacts with c-Rel to Mediate NF-κB Repression

**DOI:** 10.1371/journal.pone.0018670

**Published:** 2011-04-07

**Authors:** Louiza Loizou, Kristian G. Andersen, Alexander G. Betz

**Affiliations:** 1 MRC Laboratory of Molecular Biology, Cambridge, United Kingdom; 2 Center for Systems Biology, Department of Organismic and Evolutionary Biology, Harvard University, Cambridge, Massachusetts, United States of America; 3 Broad Institute, Cambridge, Massachusetts, United States of America; Agency for Science, Technology and Research (A*STAR), Singapore

## Abstract

Expression of the lineage-specific DNA-binding factor Foxp3 controls the development and function of naturally occurring regulatory T cells. Foxp3 has been shown to interact with a multitude of transcriptional regulators including NFAT, NF-κB (p65), Runx1 and RORγt, as well as the histone modification enzymes TIP60, HDAC7 and HDAC9. The sum of these interactions is believed to cause the change in the transcriptional program of regulatory T cells. Here we show that Foxp3 directly or as part of a multimeric complex engages with the NF-κB component c-Rel. We demonstrate that the N-terminal region of Foxp3 is required for the binding of c-Rel, but not NFAT. Conversely, deletion of the forkhead domain causes a loss of interaction with NFAT, but not c-Rel. Our findings are of particular interest, as c-Rel is crucial for the induction of Foxp3 in regulatory T cells during thymic development, but has to be repressed in mature regulatory T cells to maintain their suppressive phenotype.

## Introduction

The NF-κB family of transcription factors is coordinating the expression of a wide variety of genes [1]. The expression of its family member c-Rel is restricted to the haematopoietic and lymphoid cell lineages. In T cells, it is an important mediator of pro-inflammatory gene activation [1]. T cell receptor (TCR) signaling in combination with CD28 co-stimulation leads to the activation of c-Rel, resulting in the up-regulation of a variety of pro-inflammatory cytokines such as IL-6, IL-12, IL-15 and IFN-γ [2]. Furthermore, c-Rel plays a key role in the transcriptional activation of IL-2 [3], [4]. Indeed, one of the defining features of c-Rel deficient mutant mice is the severe abrogation of IL-2 expression in the T cell compartment [5]. Somewhat surprisingly, c-Rel also appears to play a central role in the development of regulatory T (T_R_) cells [6]–[12]. TCR signaling in thymic T cells results in the formation of a c-Rel enhanceosome at the Foxp3 promoter. Foxp3 is exclusively expressed in regulatory T (T_R_) cells and is considered to be a master regulator of T_R_ cell function [13], [14]. It was initially thought to be a transcriptional repressor [15], but it is now generally believed to work as both a repressor and an activator [16]–[18]. It plays a key role in the development and function of T_R_ cells [19]–[21]. FOXP3 mutants in human patients lack functional, thymically derived CD4^+^CD25^+^ regulatory T_R_ cells and develop a severe systemic immune dysregulation, polyendocrinopathy, enteropathy, X-linked syndrome (IPEX) [22]. While lack of Foxp3 leads to a deficiency in functional T_R_ cells, ectopic expression of the gene in conventional T_H_ cells leads to a gain of T_R_ cell phenotype [19], [20].

The fact that the c-Rel is involved in both, the pro-inflammatory transcriptional program and the opening of the Foxp3 locus required for T_R_ cells development appears to be paradoxical. Indeed, over-expression of c-Rel in T_R_ cells handicaps their suppressive activity and function [7]. This suggests that whilst c-Rel is required for the development of T_R_ cells through activation of Foxp3 transcription, its pro-inflammatory activities have to be repressed in mature T_R_ cells. The fact that Foxp3 exerts its function by interacting with a variety of transcription factors such as NFAT [18], [23], Runx1 [24] and RORγt [25], [26], all of which play important roles in the development and function of conventional T_H_ cells, makes it a prime candidate for the suppression of c-Rel. Here we show that Foxp3 interacts with c-Rel and thereby represses c-Rel mediated NF-κB activation.

## Results

### Foxp3 inhibits c-Rel mediated NF-κB activation

**Figure 1:**
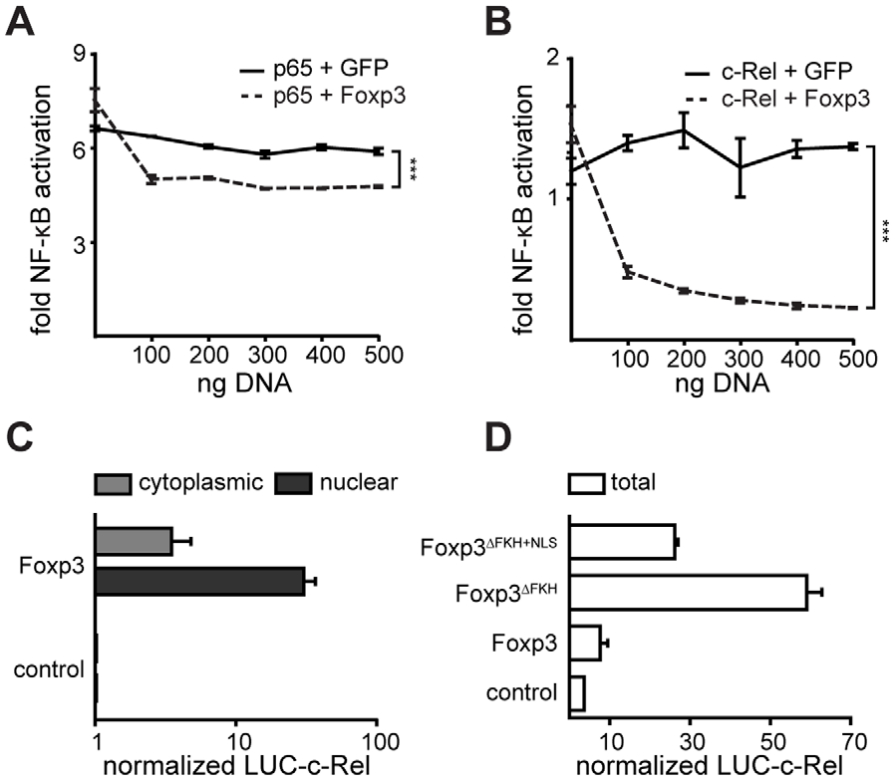
Foxp3 inhibits c-Rel mediated NF-κB activity. (A-B) 293ET cells were transfected with 400 ng of (A) m5p[FLAG-p65] or (B) m5p[FLAG-c-Rel] together with 50 ng of the NF-κB-reporter (firefly) m3p-luc[NF-κB], 20 ng pRL-TK and increasing amounts of P8[HA-Foxp3] or GFP as a control gene (P8[control]) as indicated. Forty-eight hours later, the cells were analyzed for NF-κB activity, which was normalized against the renilla luciferase signal. Transfections were performed in duplicate wells and two samples were measured in each case (*** = *p*<0.001, ANOVA). (C) 293ET cells were co-transfected with m5p[luc-c-Rel] and m5p[FLAG-Foxp3] or GFP as a control gene m5p[FLAG-control]. Forty-eight hours later cytoplasmic and nuclear cell lysates were prepared and the interaction between the two proteins were measured using LUMIER assays. (D) 293ET cells were co-transfected with m5p[luc-c-Rel] and m5p[FLAG-Foxp3^mut^] or FLAG-GFP as a control gene m5p[FLAG-control]. Forty-eight hours later total cell lysates were prepared and used for LUMIER assays. (A–D) In each case the experiment was repeated three times and a representative example is shown. The error-bars represent the SEM.

To assess the role of Foxp3 in the suppression of c-Rel, we transiently transfected 293ET cells with plasmids encoding (i) an NF-κB-reporter m3p-firluc[NF-κB], (ii) an internal control reporter pRL-TK, (iii) FLAG-tagged c-Rel (m5p[FLAG-c-Rel]) or p65 (m5p[FLAG-p65]) and (iv) increasing amounts of HA-tagged Foxp3 (P8[HA-Foxp3]) or GFP as a control gene (P8[control]). The resulting activity of firefly luciferase from the NF-κB-reporter was normalized to the activity of renilla luciferase arising from the control reporter. 48 h after transfection with m5p[FLAG-p65] an ∼8-fold induction of NF-κB activation was observed, compared to cells transfected with P8[control] (Fig. 1A). Transfection with m5p[FLAG-c-Rel] resulted in a ∼2-fold induction of NF-κB activation (Fig. 1B). To investigate the effect of Foxp3 on NF-κB activation, we co-transfected increasing amounts of P8[HA-Foxp3] or P8[control]. p65-mediated NF-κB activation was only mildly affected by co-expression of Foxp3 (Fig. 1A), whereas c-Rel-mediated NF-κB activation was severely inhibited (Fig. 1B). The difference in susceptibility to Foxp3-mediated repression remained even when we lowered the amount of m5p[FLAG-p65] used for the transfection (Fig. S1). While Foxp3 appears to affect both p65 and c-Rel mediated NF-κB activation, the effect on the latter is substantially more marked.

### Foxp3 interacts with c-Rel

Next, we investigated whether the observed Foxp3-mediated inhibition of NF-κB activity might be due to Foxp3 binding to c-Rel. Using LUMIER assays with lysates prepared from 293ET cells transiently transfected with m5p[FLAG-Foxp3] and m5p[luc-c-Rel], we were able to detect an interaction between Foxp3 and c-Rel, which was more marked in nuclear than cytoplasmic extracts (Fig. 1C). Removal of the FKH domain (Foxp3^ΔFKH^) led to a substantial increase in the interaction detected in crude lysates, which could be counteracted by the addition of a nuclear localization signal (Foxp3^ΔFKH+NLS^) to Foxp3^ΔFKH^ (Fig. 1D). Together the data suggest that Foxp3, directly or as part of a multimeric complex interacts with c-Rel in a FKH independent fashion. Indeed, deletion of the FKH domain leads to an increase in the amount of protein in the cytoplasm and thus appears to facilitate the interaction between the proteins.

### Regions of Foxp3 required for interaction with c-Rel

**Figure 2:**
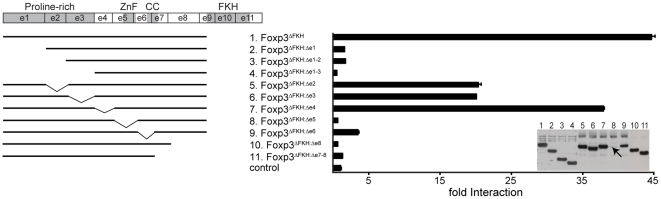
Regions of Foxp3 required for the interaction with c-Rel. 293ET cells were co-transfected with m5p[luc-c-Rel] and various m5p[FLAG-Foxp3^ΔFKH:mut^] as shown in the schematic on the left. Forty-eight hours later total cell lysates were prepared and used in LUMIER assays. Cell lysates were precipitated using anti-FLAG agarose. Fold binding indicates the ratio of luciferase activity in precipitates and cell lysates normalized to a control (GFP) reaction. Total cell lysates were analyzed by Western Blots (insert) for the expression of the FLAG-tagged Foxp3^ΔFKH:mut^ proteins. Foxp3^ΔFKH: Δe5^ is only expressed at very low levels (arrow). A representative example of three independent experiments is shown. The error-bars represent the SEM within the experiment. ZnF  =  zinc finger, CC  =  coiled-coil.

To further characterize the interaction between Foxp3 and c-Rel we performed a mutational analysis of Foxp3, assessing the domains required for the interaction between the two proteins. As before, we used LUMIER assays with total cell lysates prepared from 293ET cells transiently transfected with the various Foxp3 mutants (m5p[FLAG-Foxp3^mut^]) and m5p[luc-c-Rel]. As we had already established that the FKH domain was dispensable for binding to c-Rel, we based the various deletion and point mutants on Foxp3^ΔFKH^ (Fig. 2). Deletion of exon 1 (Foxp3^ΔFKH: Δe1^) was sufficient to almost entirely abrogate c-Rel binding (Fig. 2). Deletion of exon 6, which contains a coiled-coil (Foxp3^ΔFKH: Δe6^), abolished binding altogether, suggesting that dimerization of Foxp3 is required. In contrast, individual deletions of exons 2 or 3 only partially reduced binding. No interaction between Foxp3^ΔFKH: Δe5^ and c-Rel could be detected, but this particular mutant was expressed poorly (Fig. 2, arrow) making an interpretation of this result all but impossible. Deletion of exon 8 (Foxp3^ΔFKH: Δe8^), which has been reported to be involved in Runx1 binding [24], also led to a loss of interaction between c-Rel and Foxp3.

### The transactivation domain of c-Rel is required for interaction with Foxp3

c-Rel, like p65, belongs to the NF-κB family of proteins, which are defined by highly conserved N-terminal DNA-binding domains (REL homology domains; RHD) [1]. They are sequestered in the cytoplasm as homo- or heterodimers through association with IκB, which binds to their IPT (Ig-like-plexins-transcription factors) domains [27], [28]. The C-termini of c-Rel and p65 contain non-conserved transactivation domains, which interact with various components of the basal transcription apparatus [28], [29].

**Figure 3:**
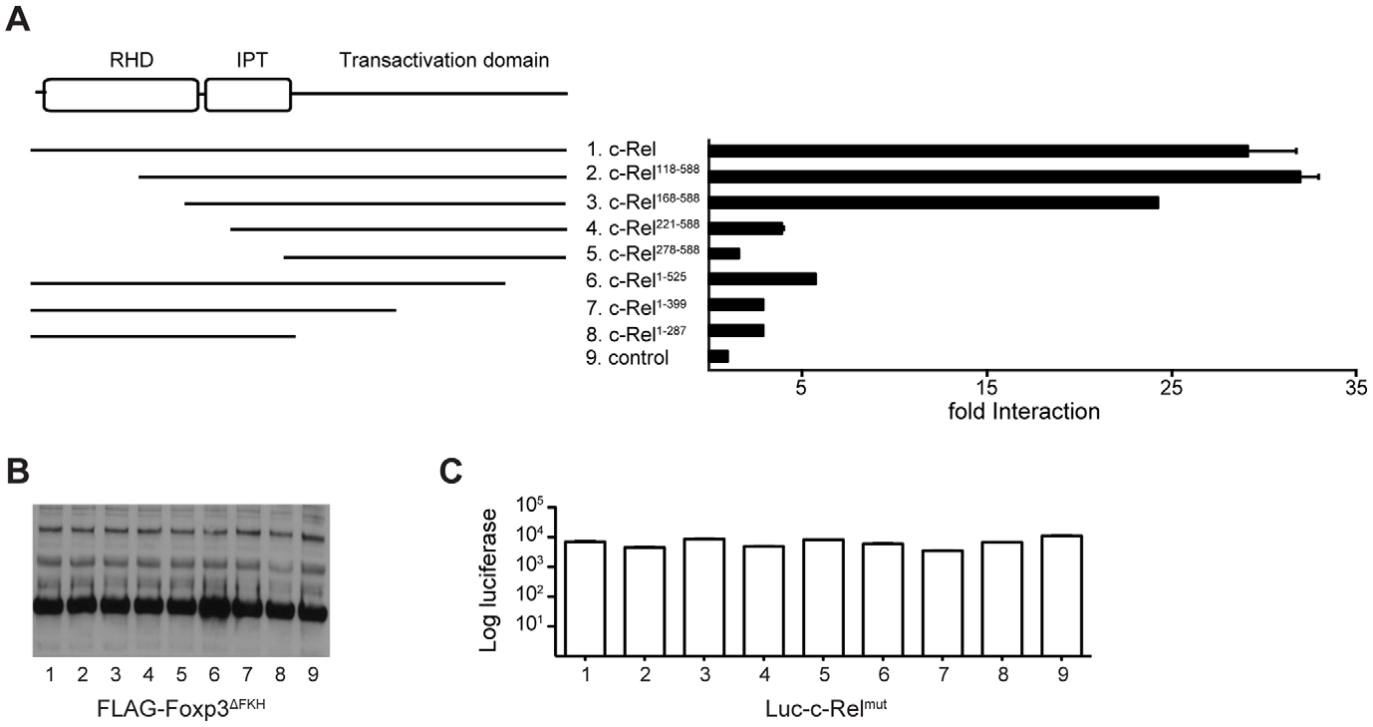
The C-terminal transactivation domain of c-Rel is required for the interaction with Foxp3. (A–C) 293ET cells were co-transfected with m5p[FLAG-Foxp3^ΔFKH^] and various m5p[luc-c-Rel^mut^] as shown in the schematic on the left. Forty-eight hours later total cell lysates were prepared and used in LUMIER assays. Cell lysates were precipitated using anti-FLAG agarose. (A) Fold binding indicates the ratio of luciferase activity in precipitates and cell lysates normalized to a control (GFP) reaction. (B) Total cell lysates were analyzed by Western Blots (insert) for the expression of the FLAG-tagged Foxp3^ΔFKH^ protein. (C) Total luciferase activity in lysate to show the expression of luc-c-Rel^mut^. A representative example of three independent experiments is shown. The error-bars represent the SEM within the experiment.

To map the regions of c-Rel required for Foxp3 binding, we performed LUMIER assays with lysates prepared from 293ET cells transiently transfected with m5p[FLAG-Foxp3^ΔFKH^] and various c-Rel mutants (m5p[luc-c-Rel^mut^]). Deletion of the N-terminal 167 amino acids (c-Rel^118-588^) did not affect the interaction of c-Rel with Foxp3^ΔFKH^, indicating that the RHD is not required for the interaction (Fig. 3). However, if the deletions extended to the IPT domain (c-Rel^221-588^ and c-Rel^278-588^) the interaction with Foxp3^ΔFKH^ was abolished. Likewise, a C-terminal deletion of the last 63 amino acids of c-Rel (c-Rel^1-525^) was sufficient to severely compromise binding to Foxp3^ΔFKH^ and further deletion of the transactivation domain (c-Rel^1-399^ and c-Rel^1-287^) completely abolished the interaction between the two proteins (Fig. 3). This suggest, that c-Rel binds to Foxp3 via its C-terminal transactivation domain and the loss of binding in the absence of the entire RHD and IPT indicates that dimerization of c-Rel is required.

### c-Rel binding to Foxp3 is not required for its interaction with NFAT

**Figure 4:**
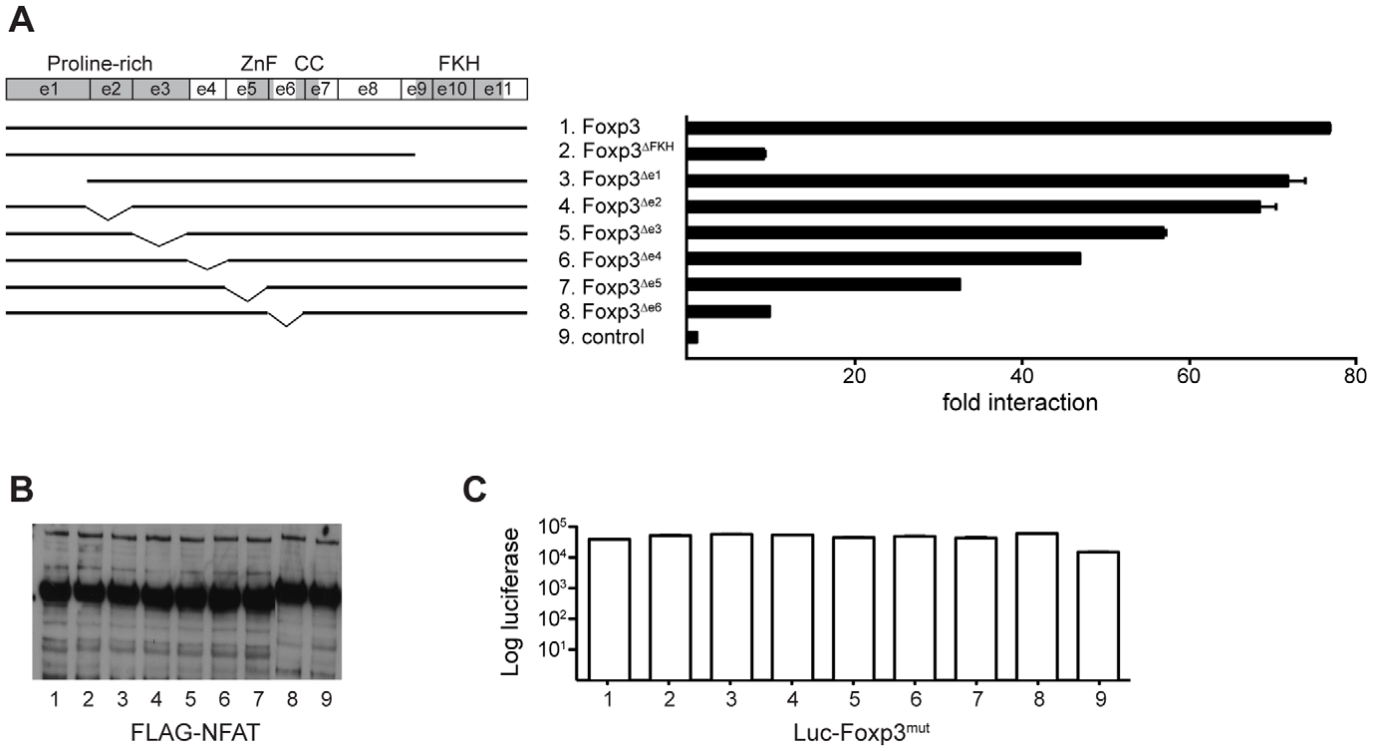
The forkhead domain of Foxp3 interacts with NFAT. (A–C) 293ET cells were co-transfected with m5p[FLAG-NFAT] and various m5p[luc-Foxp3^mut^] as shown in the schematic on the left. Forty-eight hours later total cell lysates were prepared and used in LUMIER assays. Cell lysates were precipitated using anti-FLAG agarose. (A) Fold binding indicates the ratio of luciferase activity in precipitates and cell lysates normalized to a control (GFP) reaction. (B) Total cell lysates were analyzed by Western Blots for the expression of the FLAG-tagged NFAT proteins. (C) Total luciferase activity in lysate to show the expression of luc-Foxp3^mut^. A representative example of three independent experiments is shown. The error-bars represent the SEM within the experiment.

Foxp3 has been described to form a complex with NFAT via its FKH domain [18]. Mutations that were predicted to disrupt the interaction between the two proteins, interfered with the ability of Foxp3 to inhibit the expression of several cytokines, including IL-2, and to up-regulate T_R_ cell associated markers such as CD25 and CTLA-4 [18]. However, an N-terminal deletion mutant of Foxp3 was also incapable of performing these functions, suggesting that binding of further factors might be required [18]. It remained unclear whether this is facilitated through interaction with a different region of NFAT or whether this is independent of NFAT [18]. To test whether the N-terminal region of Foxp3 is involved in NFAT binding, we performed a mutational analysis of Foxp3 assessing the domains required for this interaction. As previously shown [18], we found that deletion of the FKH domain (Foxp3^ΔFKH^) abrogated NFAT binding (Fig. 4). In contrast, deletion of exon 1 (Foxp3^Δe1^) had no effect on the interaction between the two proteins. As was the case for c-Rel binding, the deletion of the coiled-coil of Foxp3 (Foxp3^Δe6^) had a marked effect on NFAT binding. Individual deletions of all other exons led only to a partial reduction in the interaction between Foxp3 and NFAT (Fig. 4). In agreement with previous studies [18], this shows that the FKH is required for NFAT binding, whereas the N-terminal region is dispensable for complex formation between the two proteins. Furthermore, this indicates that the interaction of Foxp3 with c-Rel can occur in the absence of its interaction with NFAT and vice versa.

### The N-terminal region of Foxp3 is required for NF-κB repression

**Figure 5:**
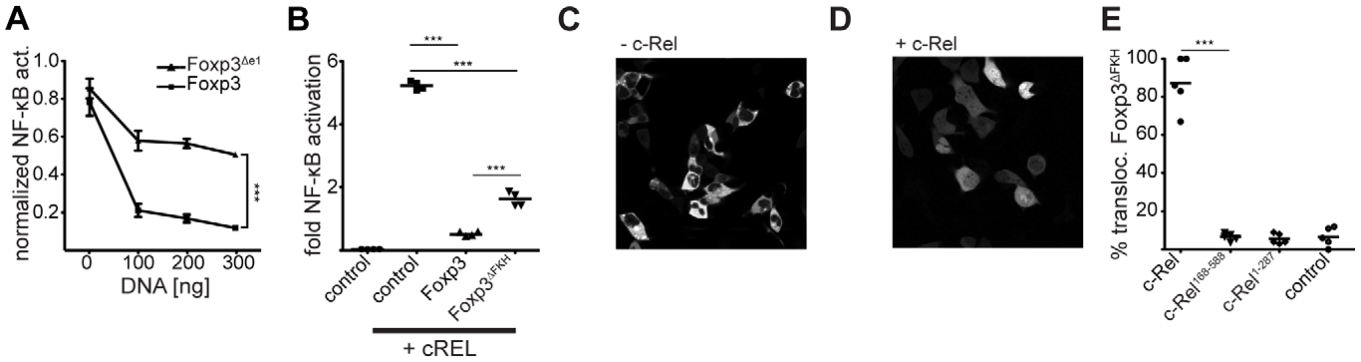
c-Rel causes a shuttling of Foxp3^ΔFKH^ **into the nucleus.** (A) 293ET cells were transfected with 400 ng m5p[c-Rel], 50 ng m3p-luc[NF-κB], 20 ng pRL-TK and increasing amounts of P8[Foxp3] or P8[Foxp3^Δe1^]. Two independent experiments were performed and fold NF-κB activation was pooled and normalized against the highest signal. The error-bars represent the SEM, (*** = *p*<0.001; two-way ANOVA). (B) Alternatively, the cells were transfected with 50 ng m3p-luc[NF-κB], 20 ng pRL-TK and 400 ng P8[control] (GFP), P8[Foxp3] or P8[Foxp3^ΔFKH^] in the presence or absence of 400 ng m5p[c-Rel]. Forty-eight hours later, the cells were analyzed for NF-κB activity, which was normalized against the renilla luciferase signal (*** = *p*<0.001; unpaired *t* test). (C-E) 293ET cells were co-transfected with m5p[GFP-Foxp3^ΔFKH^] in the (C) absence or (D) presence of m5p[c-Rel] and analyzed by confocal microscopy after 48 h. (E) Pooled data from two independent experiments where 293ET cells were co-transfected with m5p[GFP-Foxp3^ΔFKH^] and rel-homology domain deletion (c-Rel^168-588^) or transactivation domain deletion (c-Rel^1-287^) of c-Rel. For each of the conditions, several randomly picked field of visions (∼10 cells) were scored for the presence of Foxp3^ΔFKH^ in the nucleus. As a control, cells were transfected with rCD8α (m5p[control]) (*** = *p*<0.001; unpaired *t* test).

Our data suggests that the N-terminal region plays a crucial role in c-Rel binding, but is not required for NFAT interaction, which binds to the FKH domain. Thus, we investigated the role of these domains in repressing c-Rel mediated NF-κB activity. We transiently transfected 293ET cells with the NF-κB reporter plasmid and the relevant Foxp3 mutant, as described above. Deletion of exon 1 (Foxp3^Δe1^), which disrupts c-Rel binding (Fig. 2), led to a marked, albeit not complete, reduction in the repression of c-Rel induced NF-κB activation (Fig. 5A). Disruption of NFAT binding through the deletion of the FKH domain (Foxp3^ΔFKH^) had only a minor effect on the potency of the repression of c-Rel mediated NF-κB activation (Fig. 5B).

### c-Rel causes a shuttling of Foxp3^ΔFKH^ into the nucleus

We were intrigued to find that Foxp3^ΔFKH^, despite having lost its nuclear localization [30], was still capable of regulating c-Rel mediated NF-κB activation. As Foxp3 is a DNA binding factor, we would have expected that nuclear localization is a prerequisite for its activity as a transcriptional regulator [31]. We hypothesized that either Foxp3^ΔFKH^ retains c-Rel in the cytoplasm or c-Rel shuttles Foxp3^ΔFKH^ into the nucleus. To test this, we transfected 293ET cells with plasmids encoding c-Rel (m5p[c-Rel]) and a GFP-tagged Foxp3^ΔFKH^ (m5p[GFP-Foxp3^ΔFKH^]) and analyzed the sub-cellular localization of GFP-Foxp3^ΔFKH^ by confocal microscopy. In 293ET cells a large proportion of ectopically expressed c-Rel localizes to the nucleus in the absence of any kind of stimulation (data not shown). Whilst in the absence of c-Rel, GFP-Foxp3^ΔFKH^ was predominantly found in the cytoplasm (Fig. 5C), co-expression of c-Rel caused a redistribution of GFP-Foxp3^ΔFKH^ into the nucleus (Fig. 5D). Notably, deletion of either the RHD (c-Rel^168-588^) or transactivation domain (c-Rel^1-287^) of c-Rel led to a loss of this redistribution (Fig. 5E). The former causes a loss of nuclear translocation of c-Rel [1], whereas the latter leads to a loss of Foxp3 binding (Fig. 3). Wild type Foxp3 is already nuclear by itself and co-transfection of c-Rel didn't change this (data not shown).

## Discussion

Here we show that Foxp3 either directly or as part of a larger multimeric complex [32] interacts with c-Rel. We found that deletion of the N-terminal region of Foxp3 led to a loss of inhibition of c-Rel-mediated NF-κB activity. Furthermore, we demonstrate that this region is required for binding of the transactivation domain of c-Rel, which interacts with components of the basal transcription apparatus such as TATA-binding proteins, p300 and cyclic-AMP-response element (CREB)-binding protein (CBP) [33]. An attractive interpretation would be that Foxp3 competes with such factors for binding, which would result in an inhibition of transcription. However, simple competition for interaction with c-Rel is unlikely to be the entire explanation, as while Foxp3^ΔFKH^ is still capable of binding c-Rel, it has lost the ability to inhibit IL-2 expression [18].

In the case of IL-2 and several other Foxp3 target genes, NFAT is an essential component of transcriptional control [18]. In the absence of Foxp3, both c-Rel and NFAT activate IL-2 transcription [34], whereas in the presence of Foxp3 both appear to be involved in its repression. Foxp3^Δe1^ binds NFAT, but has lost its ability to interact with c-Rel. Conversely Foxp3^ΔFKH^ interacts with c-Rel, but not NFAT. This demonstrates that either factor can bind to Foxp3 without the other being present, suggesting that in some cases the individual interactions might be sufficient for the regulation of target gene transcription. However, we observed an increase in the binding of c-Rel to Foxp3 when we deleted its FKH and thereby interfered with NFAT binding. However, as this also leads to a repositioning of Foxp3 into the cytoplasm, an experimental bias favoring an interaction with c-Rel might be introduced. An alternative interpretation would be that under physiological conditions c-Rel and NFAT directly or indirectly compete for binding to Foxp3.

While we found that c-Rel can shuttle Foxp3^ΔFKH^ into the nucleus, this might not always be sufficient to reconstitute its function. Indeed, it has already been shown that the FKH is crucial for nuclear localization, DNA binding as well as interaction with NFAT [18]. We consider the shuttling of Foxp3^ΔFKH^ by c-Rel to be an experimental peculiarity. Nevertheless, it points towards a potential role of c-Rel in transporting Foxp3 towards particular target genes, which warrants further investigations in future.

Foxp3 and c-Rel predominantly exist as dimers. In the case of Foxp3, this is mediated by the coiled-coil [35]. Point mutations found in IPEX patients, which lie in critical residues (ΔK249 or ΔE250) within the coiled-coil, are defective in dimerization and lead to a loss of Foxp3 function [35]. For c-Rel, the dimerization is dependent on the presence of the IPT domain [36]. We show that disruption of either the coiled-coil in Foxp3 or IPT in c-Rel leads to a loss of complex formation between the proteins. This suggests that both Foxp3 and c-Rel have to be in a dimerized form in order to interact.

We found that while the FKH domain of Foxp3 is not necessary for c-Rel binding, deletion of exon 8, lying immediately upstream of the FKH is. This region of Foxp3 is required for its interaction with the transcription factor Runx1, which also has been implicated in IL-2 regulation [24]. However, we cannot exclude that the progressive deletion of the C-terminus of Foxp3 leads to a loss of structural integrity *per se*, rather than invoking a requirement for cooperative binding of Runx1 and c-Rel.

It has been proposed that Foxp3 forms high molecular weight, multimeric transcriptional complexes [32]. From this, and other studies [18], [23], [24], [26], [37], it is becoming increasingly clear that Foxp3 works by hijacking already resident transcriptional mechanisms that are important for T cell activation. In particular the N-terminal region has been shown to interact with many factors including RORγt, RORα [25], [26], Tip60, HDAC7/9 [37] and Eos [38]. It might well be that the composition of these complexes differs depending on the developmental stage and activation status of the cell, as well as the target gene. In some cases, the presence of Foxp3 may lead to a switch from gene activation to repression, and in others it may lead to enhanced transcription. This might explain how a single factor, Foxp3, can redirect the transcriptional program from that of a pro-inflammatory T_H_ cell to that of a suppressive T_R_ cell - two diametrically opposed immunological T cell functions.

## Materials and Methods

### Cells

293ET human embryonic kidney cells [39] were grown in IMDM supplemented with 10% FCS 50 µM β-mercaptoethanol and 50 µg/ml gentamicin. Eighteen hours prior to transfection, 293ET cells were seeded into plates at a density of 1.5×10^5^ cells/cm^2^. Transfections were performed using standard calcium phosphate precipitation.

### Plasmids

In most experiments, the low expressing plasmid m5p was used [40]. For the measurement of NF-κB activation, a 8xNF-κB-Luciferase (Firefly) reporter (m3p-firluc[NF-κB]), as well as high-expressing pEAK8-based plasmids expressing Foxp3 (P8[Foxp3]) or GFP as an irrelevant control gene (P8[control]) were used.

### NF-κB reporter assays

293ET cells were seeded and eighteen hours later the cells were transfected with 50 ng of m3p-firluc[NF-κB], 20 ng of pRL-TK control vector and the indicated plasmids. The transfected cells were analyzed 48 h later using the Dual Luciferase Reporter Assay system (Promega) following the manufacturer's instructions.

### Preparation of cytoplasmic and nuclear extracts

Preparation of 293ET extracts was performed on ice and all centrifugations were performed at 4°C. Crude lysates were prepared by addition of lysis buffer (20 mM Tris, 150 mM NaCl, 5 mM EDTA, 0.1% Triton X-100, 10% glycerol, 5 µg/ml leupeptin, 2 µg/ml aprotinin, 1 mM benzamidine, 1 µM PMSF) and incubation for 15 minutes. The extracts were cleared by centrifugation at 16000 x*g* for 15 minutes. Where cytoplasmic and nuclear extracts were required, cells were collected, washed with PBS and lysed in hypotonic buffer (10 mM HEPES pH 7.9, 10 mM KCl, 0.1 mM EDTA, 0.1 mM EGTA, 1 mM DTT, 5 µg/ml leupeptin, 2 µg/ml aprotinin, 1 mM benzamidine, 1 µM PMSF) for 5 minutes. NP-40 was added to a final concentration of 0.6%, the cells were vortexed briefly and nuclei were collected by centrifugation at 16000 x*g* for 1 minute. The nuclear pellet was vortexed for 15 minutes in cold hypertonic buffer (20 mM HEPES pH 7.9, 0.4 M NaCl, 1 mM EDTA, 1 mM EGTA, 1 mM DTT, 5 µg/ml leupeptin, 2 µg/ml aprotinin, 1 mM benzamidine, 1 µM PMSF) to release nuclear proteins. Following centrifugation at 16000 x*g* for 15 minutes, the supernatant was collected and where necessary, the protein concentration was quantified using Bradford (BIO-RAD). Unless used directly, the extracts were stored at -70°C.

### Luminescence-based mammalian interactome mapping (LUMIER)

293ET cells were co-transfected with a FLAG-tagged construct and a Renilla Luciferase-tagged construct. Extracts were incubated with anti-FLAG M2 Affinity Gel (Sigma) for 2 h, rotating at 4°C. Unbound protein was removed by washing 5 times with 20 mM Tris, 150 mM NaCl, 5 mM EDTA, 0.1% Triton X-100, 10% glycerol and centrifugation at 6000 x*g* for 30 seconds. Bound proteins were eluted from the affinity gel by incubating with 150 µg/ml FLAG peptide (Sigma) for 30 minutes, rotating at 4°C. A sample of the lysate (input) and a sample of the gel eluate (immunoprecipitated) were measured for Renilla Luciferase activity using the Renilla Luciferase Assay Kit (Promega) as described in the manufacturer's protocol. The results are shown as the ratio of immunoprecipitated versus input Renilla Luciferase activity.

### Western blots

Extracts were separated on NuPAGE 4-12% Bis-Tris gels (Invitrogen) and blotted onto nitrocellulose membrane (Whatman) as described by the manufacturer. The membrane was blocked for 1 h at room temperature with 3% milk and incubated at 4°C overnight with one of the following antibodies: 1∶1000 of rabbit anti-c-Rel (sc-71, Santa Cruz Biotechnology), 1∶5000 of mouse anti-FLAG M2 (Sigma), 1∶1000 of mouse anti-HA.11 (Covance Research Products). The membrane was washed and incubated with anti-mouse or anti-rabbit HRP-conjugated secondary antibody (DakoCytomation) for 2 h at room temperature. HRP was detected by incubation of the membrane with ECL Detection Reagents (Amersham Biosciences).

### Translocation of Foxp3^ΔFKH^

293ET cells were co-transfected with m5p[GFP-Foxp3^ΔFKH^] in the presence or absence of m5p[c-Rel]. Twenty-four hours later, 1×10^4^ cells were transferred into Lab-Tec chambered coverslips (Nalge Nunc International). After an additional 24 h, the sub-cellular localization was assessed by observing for cytosolic and nuclear presence of GFP-Foxp3^ΔFKH^ by using a BioRad Radiance 2000 confocal microscope.

## Supporting Information

Figure S1 **Foxp3-mediated repression of NF-κB activity.** 293ET cells were transfected with 400 ng of m5p[FLAG-c-Rel] or 100 ng of m5p[FLAG-p65] together with 50 ng of the NF-κB-reporter (firefly) m3p-luc[NF-κB], 20 ng pRL-TK and increasing amounts of P8[HA-Foxp3]. Forty-eight hours later, the cells were analyzed for NF-κB activity, which was normalized against the renilla luciferase signal.
(TIF)
