# Supplementary figures and images for: Foxp3 Interacts with c-Rel to Mediate NF-κB Repression
Source: PLoS One. 2011 Apr 7;6(4):e18670. doi: 10.1371/journal.pone.0018670 (PMC3072406; doi:10.1371/journal.pone.0018670)

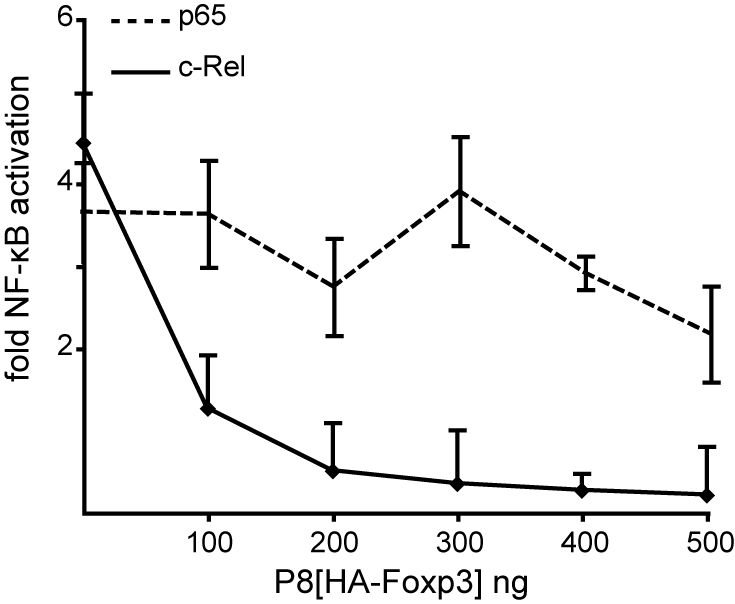

Supplement: Figure S1 — Foxp3-mediated repression of NF-κB activity. 293ET cells were transfected with 400 ng of m5p[FLAG-c-Rel] or 100 ng of m5p[FLAG-p65] together with 50 ng of the NF-κB-reporter (firefly) m3p-luc[NF-κB], 20 ng pRL-TK and increasing amounts of P8[HA-Foxp3]. Forty-eight hours later, the cells were analyzed for NF-κB activity, which was normalized against the renilla luciferase signal. (TIF) [file pone.0018670.s001.tif]
